# Molecular Insights into the Kinetic Aging Mechanisms of SBS-Modified Asphalt

**DOI:** 10.3390/ma18214821

**Published:** 2025-10-22

**Authors:** Yunjing Nie, Ye Bai, Fang Liu, Pengfei Li, Zhidong Zhou

**Affiliations:** 1 College of Civil Engineering, Taiyuan University of Technology, Taiyuan 030024, China; 2 Shanxi Jinyang Expressway Reconstruction and Expansion Project Management Co., Ltd., Jincheng 048008, China; 3 Department of Civil and Environmental Engineering, Washington State University, Pullman, WA 99164-2910, USA

**Keywords:** SBS-modified asphalt, aging, kinetic mechanism, first aging stage, second aging stage

## Abstract

The aging of SBS-modified asphalt (SBSMA) is a kinetic process that significantly deteriorates pavement performance and shortens service life. Although previous studies have explored the evolution of SBSMA during aging, the underlying kinetic mechanisms remain unclear. In this study, SBSMA samples were subjected to varying degrees of aging to simulate the kinetic aging process. Changes in four components and chemical functional groups were characterized, supporting the construction of molecular models at different aging stages. Molecular dynamics simulations indicate that the oxidation rate of SBSMA and degradation rate of SBS molecular chains are significantly higher in the initial aging stage than later, leading to a pronounced increase in cohesive energy density and solubility parameters, along with a decrease in surface free energy, fractional free volume, and binding energies, predominantly occurring during the first aging stage. Aging also shortens intermolecular distance between asphaltene molecules while increasing the distances between asphaltene–resin and asphaltene–SBS. The adsorption competition between asphaltene and SBS for lightweight components intensifies initially, whereas asphaltene exhibits stronger adsorption in the later aging stage. Furthermore, the diffusion coefficients of asphaltene and SBS increase rapidly initially then slow, causing a corresponding rapid initial decline followed by decrease in resin, aromatic, and saturate components.

## 1. Introduction

Styrene–butadiene–styrene (SBS)-modified asphalt (SBSMA) is a critical material for building durable, high-performance pavements that can withstand the increasing demands of modern traffic and varying climate conditions. Its widespread application in high-grade pavement stems from the exceptional enhancement it provides to the asphalt binder, significantly improving its elasticity, resistance to permanent deformation at high temperatures, and flexibility at low temperatures. However, SBSMA is prone to aging under conditions such as heat, oxygen, and light during its storage, transportation, paving, and long-term usage. This aging phenomenon is not merely a chemical curiosity but a primary factor leading to the premature failure of pavement structures. Aging increases the brittleness and hardness of SBSMA, reducing pavement tensile strength and leading to crack formation and other distresses [1,2,3]. These cracks allow water and other harmful agents to infiltrate the pavement structure, accelerating its deterioration. Additionally, aging weakens the adhesion between SBSMA and aggregates, causing stripping and raveling, which further compromises pavement performance and shortens service life. Therefore, a deep understanding of the aging mechanism of SBSMA is not just academically interesting but is essential for developing effective anti-aging technologies to extend pavement service life and enhance the sustainability of transportation infrastructure.

Extensive research has focused on characterizing the aging behavior of SBSMA by analyzing changes in microscopic properties and morphology. Fourier transform infrared spectrometer (FTIR) studies reveal that aging increases polar functional groups (e.g., sulfoxide and carbonyl) in SBSMA, intensifying intermolecular interactions and hardening the material [4,5]. Fluorescence microscopy (FM) observations show a reduction in SBS particle area, indicating polymer degradation and disruption of its three-dimensional network structure [6,7]. Atomic force microscopy (AFM) studies demonstrate that aging promotes the formation of bee-like structures in SBSMA and diminishes the modifying effect of SBS on the asphalt matrix [8]. The aging process of SBSMA is very complex, involving simultaneous aging of both the SBS polymer and asphalt matrix, as well as their interactions. SBS undergoes chain scission and degrades into smaller molecules, leading to the collapse of its network structure [9,10,11,12,13]. Concurrently, lightweight components in the asphalt matrix convert into heavyweight components [14]. Due to mutual protection between SBS and the asphalt matrix, their individual aging rates are slower than when aged separately [15,16]. Despite these advances, the fundamental aging mechanism of SBSMA remains incompletely understood, necessitating investigation at the molecular or atomic scale.

Molecular dynamics (MD) simulations have emerged as a powerful tool for studying SBSMA at the molecular level, enabling insights into agglomeration behavior, compatibility, and modification mechanisms [17,18,19,20,21,22,23]. Recent MD studies on SBSMA aging have revealed key findings: Yu et al. [24] observed stronger interactions between asphaltenes and lightweight components in aged SBSMA, linking sulfoxide-group formation to increased asphaltene aggregation and colloidal-structure disruption. Further, Yu et al. [25] demonstrated that SBS degradation with aging significantly reduces adhesion energy between SBSMA and aggregates. Xu et al. [26] reported that asphalt-matrix aging contributes more to viscosity increases than SBS aging, and Hu et al. [27] found that SBS, due to its unsaturated C=C bonds, ages faster initially but slower in later stages compared to the asphalt matrix. Despite these contributions, molecular-scale studies on SBSMA aging remain limited.

Notably, aging is a kinetic process with distinct stages. Yan et al. [28] observed that SBS degradation predominates in early aging stages, while Zhao et al. [29] reported rapid initial degradation followed by slower progression. Tan et al. [30] proposed a three-stage aging model: (1) slow aging with retained SBS crosslinking, (2) partial network damage, and (3) complete structural breakdown. However, the kinetic mechanisms driving these stages remain unclear. To address this gap, this study investigates the kinetic aging mechanism of SBSMA at the molecular scale, providing a foundation for improving anti-aging strategies and pavement longevity.

## 2. Research Plan

**Figure 1:**
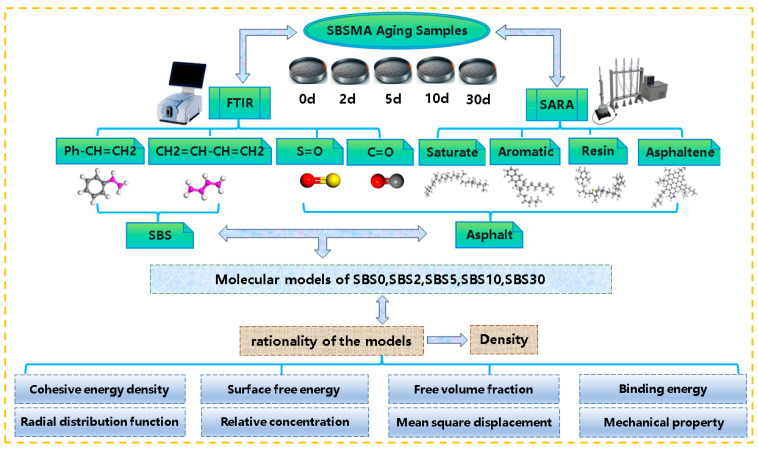
The research schematic of this study.

This study investigates the kinetic aging mechanism of SBS-modified asphalt (SBSMA) through a combined experimental and molecular dynamics (MD) simulation approach. SBSMA samples will be subjected to laboratory oven-aging to simulate different stages of the aging process. The aged samples will be systematically characterized using four-component analysis and FTIR spectroscopy, providing quantitative data on compositional changes and chemical functional-group transformations during aging. These experimental results will serve as the foundation for constructing accurate molecular models representing SBSMA at various aging stages, incorporating key parameters such as component fractions and oxidation products. Subsequently, molecular dynamics simulations will be employed to investigate the thermodynamic properties of these models, with a particular focus on analyzing the degradation kinetics of SBS polymers, and changes in asphalt-matrix properties. This study will establish a comprehensive understanding of the stage-wise aging mechanisms in SBSMA, including the relative contributions of SBS degradation and asphalt oxidation at different aging stages, ultimately providing molecular-level insights into the kinetic aging mechanism of SBS-modified asphalt. The research schematic is shown in Figure 1.

### 2.1. Materials

This study’s selected asphalt matrix was the 70# base asphalt, supplied by Shanxi Yutong Road Materials Company (Taiyuan, China). SBSMA was prepared by adding 4% SBS star-shaped polymer to the asphalt matrix, supplied by Yanshan Petrochemical Company (Beijing, China). The softening point, penetration, and ductility of asphalt matrix and SBSMA were tested separately. These technical indices are presented in Table 1, and all these indices meet the requirements of the specifications.

The study employed 70# base asphalt as the asphalt matrix, with SBSMA prepared by incorporating 4% (by weight) of star-shaped SBS polymer. The fundamental properties of both base asphalt and SBSMA were characterized as presented in Table 1, and all measured technical parameters for both the base asphalt and SBSMA satisfied the relevant specification requirements.

### 2.2. Experiments and Analysis

#### 2.2.1. Aging Tests

The aging kinetics of SBSMA were investigated through controlled thermal oxidation experiments using a forced-air oven. The experimental procedure consisted of three main steps: (1) heating the prepared SBSMA to a fluid state (160 ± 5 °C), (2) uniformly pouring approximately 10 g of the heated SBSMA into 100 mm diameter aluminum plates to achieve a consistent 1 mm thickness film, and (3) aging the samples in a forced-air oven maintained at 85 °C. Samples were retrieved after 0, 2, 5, 10, and 30 days of aging, designated as SBS0, SBS2, SBS5, SBS10, and SBS30, respectively. The mass of each sample was measured using a precision balance (to the nearest ±0.01 g) before and after the aging process to quantify mass loss due to potential volatilization. This aging protocol was designed to capture the complete kinetic profile of SBSMA degradation, from initial to advanced aging stages.

#### 2.2.2. Four-Component Separation Testing and Analysis

The evolution of SBSMA composition during aging was quantitatively analyzed using SARA (saturates, aromatics, resins, and asphaltenes) fractionation. The analysis was performed using a column chromatography system equipped with a fraction collector. Approximately 1.0 g of the SBSMA was dissolved in n-heptane and adsorbed onto 10 g of activated silica gel (80–100 mesh), which was then packed into the column as the stationary phase. The four fractions were sequentially eluted using the following solvents: (1) 150 mL of n-heptane (≥99.0%) for saturates; (2) 150 mL of toluene (≥99.5%) for aromatics; and (3) 150 mL of a toluene/ethanol (*v*/*v* = 1:1, ≥99.5%/≥99.7%) mixture for resins. The asphaltenes, defined as the n-heptane-insoluble fraction, were subsequently recovered from the silica gel by dissolution with 100 mL of dichloromethane (≥99.9%). The mass fraction of each component was determined by gravimetry. Each collected eluent was evaporated at ambient temperature and then dried in a vacuum oven at 80 °C until a constant weight was achieved. The mass of each fraction was measured, and its content was calculated as a percentage of the total initial mass of the sample. All chemical reagents were used without further purification.

These compositional changes critically influence the SBSMA’s physicochemical properties and must be accurately incorporated into molecular models to ensure their representativeness. This approach enables precise determination of component redistribution patterns, providing essential input parameters for subsequent molecular-modeling efforts.

**Figure 2:**
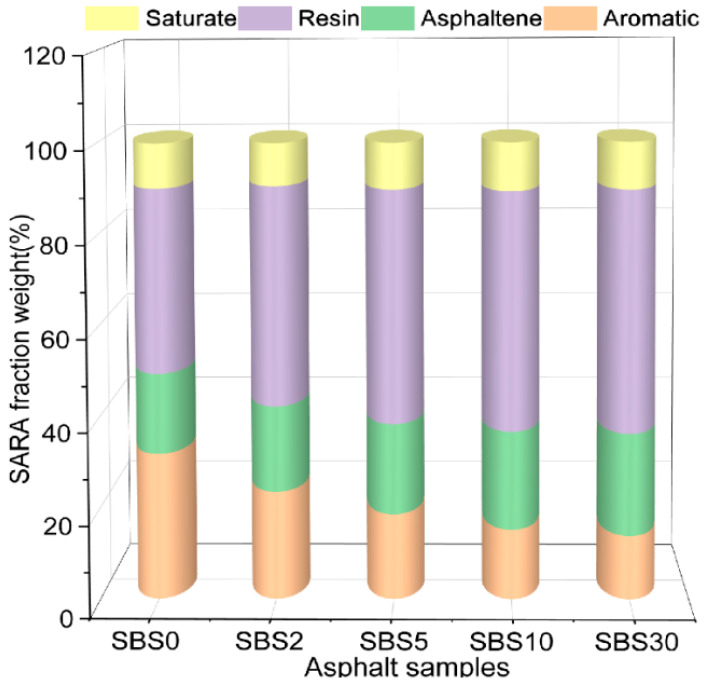
SARA mass fraction of SBSMA during kinetic aging process.

Figure 2 presents the mass fraction changes in each component throughout the aging process. Notably, while saturate content remained relatively constant, systematic changes were observed in other fractions: a progressive increase in asphaltene and resin content, accompanied by a corresponding decrease in aromatic content. The average mass loss after 30 days of aging was minimal (0.8%), indicating that volatilization was not a dominant process. To conclusively attribute the observed trends to chemical transformation rather than physical loss, the percentage changes were corroborated by analyzing the net mass change in each fraction. The analysis confirmed a net decrease in the mass of aromatics and a net increase in the mass of resins and asphaltenes. These transformations confirm the oxidative conversion of lighter aromatic components to heavier resin and asphaltene fractions, consistent with established asphalt-aging mechanisms.

#### 2.2.3. FTIR Testing and Analysis

The aging process of SBSMA is characterized by two key chemical transformations: (1) the progressive formation of oxygen-containing functional groups (carbonyl and sulfoxide) and (2) the scission of SBS polymer chains. To accurately represent these molecular-scale changes in SBSMA models, this study quantified both the oxidative aging products and polymer degradation through FTIR using a Bruker Alpha II spectrometer, equipped with a diamond attenuated total reflectance (ATR) accessory. Spectra were acquired with a resolution of 4 cm^−1^ and 16 scans per spectrum. To ensure representativeness, each sample was thermally homogenized by heating to 150 °C with gentle stirring before being deposited onto the ATR crystal. Triplicate measurements were performed on different spots for each sample, and the average spectrum was used for subsequent analysis.

**Figure 3:**
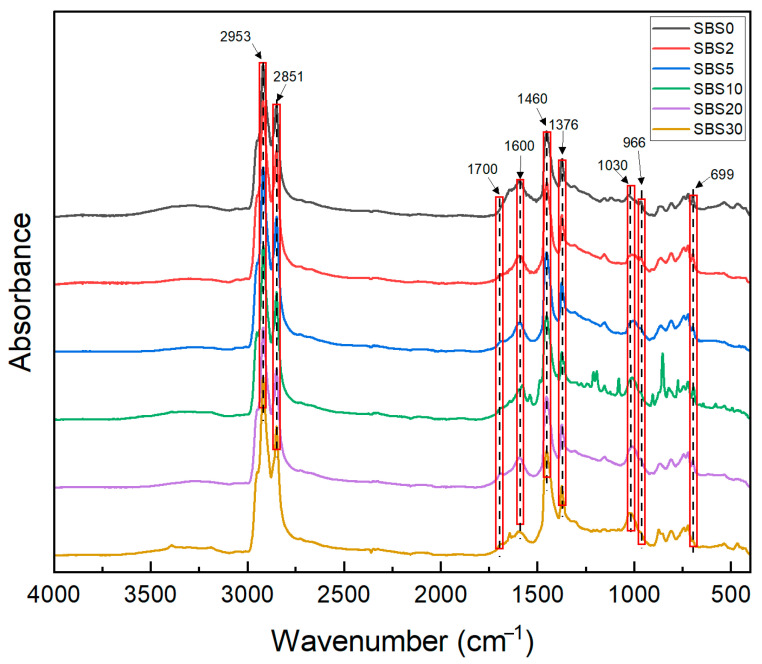
The averaged FTIR of various SBSMA samples.

The various SBSMA samples were tested, and their infrared spectra were obtained, as shown in Figure 3. A comprehensive assignment of the major observed absorption bands is provided in Table 2, which details the corresponding vibrational modes and molecular origins. The evolution of the FTIR spectra reveals two key trends: (1) progressive intensification of carbonyl (1700 cm^−1^) and sulfoxide (1030 cm^−1^) absorption bands, indicating oxidative aging [31], and (2) attenuation of characteristic SBS peaks at 699 cm^−1^ (polystyrene) and 966 cm^−1^ (polybutadiene), demonstrating polymer degradation [32]. It is noteworthy that the spectrum of the 10-day aged sample, in one of the initial measurements, exhibited several distinct but non-reproducible bands (e.g., at 1542, 1199, and 1086 cm^−1^), which could be attributed to localized heterogeneity or transient oxidation products. However, the averaged triplicate spectrum followed the overall aging trend, confirming the robustness of the main conclusions. Quantitative analysis employed four spectral indices, sulfoxide index (SI), carbonyl index (CI), polystyrene index (IS), and polybutadiene index (IB), to investigate the chemical properties of SBSMA during the kinetic aging process, according to Equations (1)–(4),

(1) $$CI=\frac{A_{1700}}{\sum A}$$

(2) $$SI=\frac{A_{1030}}{\sum A}$$

(3) $$I_{S}=\frac{A_{699}}{\sum A}$$

(4) $$I_{B}=\frac{A_{966}}{\sum A}$$

where $A_{1700}$, $A_{1030}$, $A_{699}$, and $A_{966}$ are the peak areas at the wavenumbers of 1700 cm^−1^, 1030 cm^−1^, 699 cm^−1^, and 966 cm^−1^, and ∑A is the sum of all the peak areas. To ensure reproducibility, the detailed procedure for obtaining these area values is described as follows: A consistent multi-point linear baseline correction was applied prior to integration. The specific integration limits for each peak were defined as 1720–1680 cm^−1^ for the carbonyl band (A_1700_), 1050–1010 cm^−1^ for the sulfoxide band (A_1030_), 710–680 cm^−1^ for the polystyrene band (A_699_), and 980–950 cm^−1^ for the polybutadiene band (A_966_). The total peak area (∑A) was calculated by integrating the entire spectrum from 4000 to 600 cm^−1^ after baseline correction.

**Figure 4:**
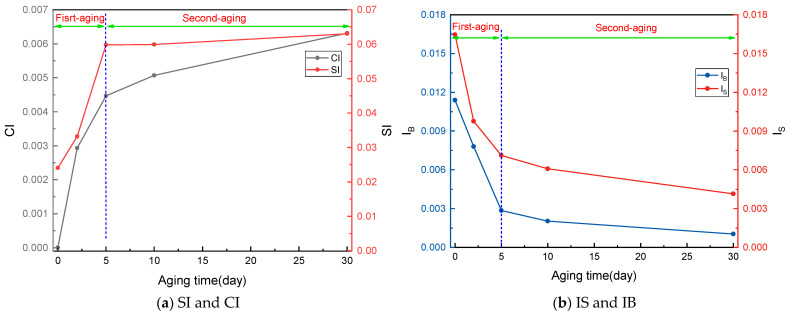
The changes in SI, CI, IS, and IB of SBSMA with aging time.

Figure 4a reveals distinct kinetic patterns in the oxidative aging of SBSMA. The SI exhibits rapid growth during the initial 0–5 day period before stabilizing, suggesting sulfur depletion as available sulfur atoms become incorporated into sulfoxide groups. Similarly, the CI shows accelerated formation in the first 5 days, followed by a reduced growth rate from 5 to 30 days. This two-phase behavior indicates a transition from primary sulfur oxidation to subsequent carbon-centered oxidation, where benzyl carbons react with oxygen to form ketone groups—a mechanism consistent with previous reports [33,34].

Correspondingly, Figure 4b demonstrates that both IS and IB indices display exponential decay patterns, with significantly faster reduction rates during the initial 0–5 day period compared to the subsequent 5–30 day period. These trends clearly indicate that SBS-polymer degradation predominantly occurs during the first aging stage, with chain scission rates decreasing substantially once the majority of vulnerable bonds have broken.

Based on these observations, we propose a two-stage aging mechanism. First aging stage (0–5 days): characterized by rapid sulfur oxidation and extensive SBS-network degradation, accounting for the majority of polymer-chain scission. Second aging stage (5–30 days): marked by slower carbon-centered oxidation and gradual continuation of SBS degradation, primarily affecting remaining stable bonds.

## 3. Molecular Models Establishment

### 3.1. Molecular Structure of SBSMA During the Kinetic Aging Process

The molecular modeling of SBSMA-aging kinetics requires careful consideration of both asphalt-matrix evolution and SBS-polymer degradation. A molecular modeling approach systematically addresses these components across five aging states (SBS0-SBS30) to capture the progressive aging mechanisms.

#### 3.1.1. Asphalt-Matrix Modeling

**Figure 5:**
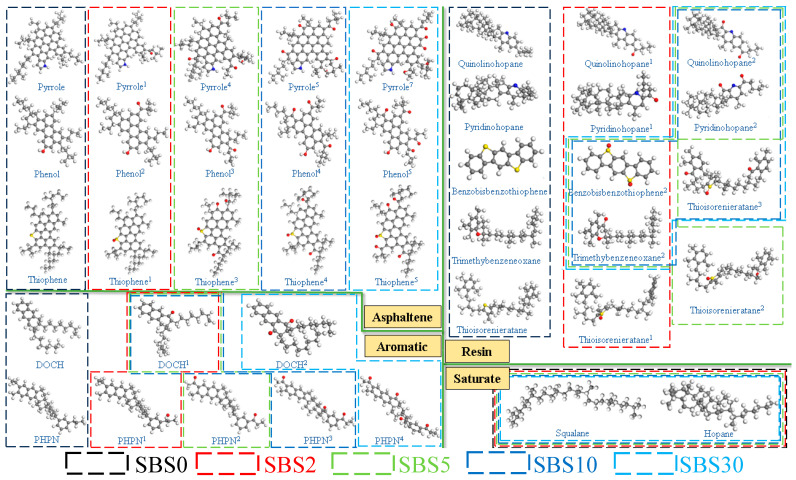
The specific asphalt matrix of SBS0–SBS30 molecular structure models (oxygen: red).

For the unaged condition (SBS0), the 12-component molecular asphalt model developed by Li and Greenfield [35] was adopted, selected for its accurate density prediction and widespread acceptance in asphalt research. The fully aged condition (SBS30) employed the oxidized model proposed by Xu and Wang [36], featuring complete conversion of sulfur to sulfoxide groups and benzyl carbons to carbonyl groups [37,38,39]. Intermediate aging states (SBS2, SBS5, SBS10) were modeled by progressively introducing oxygen atoms into the base 12-component molecular model, following two key principles: sequential oxidation, that is, sulfoxide formation precedes carbonyl-group generation, and oxidation extent that matched to experimental FTIR quantification of SI and CI indices. The molecular transformations across aging states are illustrated in Figure 5, showing the systematic progression from unaged to fully oxidized structures.

#### 3.1.2. SBS-Polymer Modeling

**Figure 6:**
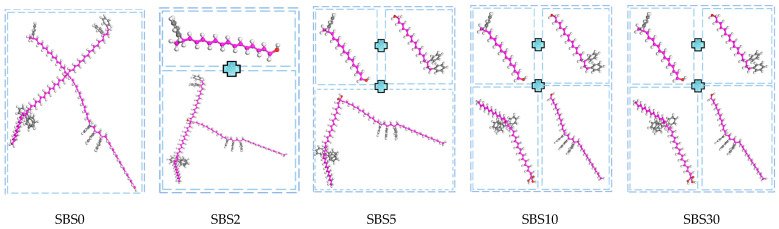
The specific SBS of SBS0-SBS30 molecular structure models.

The star-shaped SBS polymer (30% styrene, 70% butadiene) undergoes progressive chain scission during aging. The modeling approach captures this degradation: (1) initial state (SBS0) maintains the intact star-shaped polymer structure; (2) final state (SBS30) represents complete fragmentation into four segments [38]; and (3) intermediate states exhibit controlled chain scission proportional to FTIR-derived IS and IB indices. The degradation mechanism focuses on cleavage of vulnerable C=C bonds in butadiene and styrene units [16,40,41], with resultant formation of oxygen-containing terminal groups (-OH, -COOH) as observed experimentally [1,42]. Figure 6 illustrates the progressive fragmentation patterns of SBS.

### 3.2. Molecular Number of SBSMA During the Kinetic Aging Process

The molecular number of each aging state of SBS0–SBS30 were rigorously optimized to reflect three critical aspects of aging-induced changes: SARA fraction evolution, ensuring alignment with four-component separation test results; oxidative group incorporation, matching FTIR-derived SI and CI values; SBS degradation extent, correlating with combined IS and IB indices. Through constrained optimization algorithms, the precise molecular numbers for each aging state of SBS0–SBS30 (Table 3) were determined, to achieve the accurate representation of component content changes, properly accounting for oxidative functional-group formation, and realistic simulation of polymer degradation kinetics.

### 3.3. Molecular Dynamics Simulation Protocol

The molecular models of SBSMA at various aging stages (SBS0 to SBS30) were constructed and simulated. The simulations were performed based on the molecular compositions detailed in Table 3 and followed a multi-step protocol to ensure the models reached realistic and equilibrated configurations. The entire process is described in detail below.

(1) Model Construction and Energy Minimization: Molecular structures of individual components (asphalt molecules and SBS fragments) were first built and geometrically optimized using the Forcite module. This initial energy minimization, employing the Smart Minimizer algorithm with a convergence tolerance of 10^−4^ kcal/mol, ensured each molecule started from a stable local energy minimum.
(2) Amorphous Cell Assembly: The initial three-dimensional periodic simulation cells for each SBSMA system were constructed using the Amorphous Cell module. The models were built based on the specified number of molecules from Table 3, targeting an initial low density of 0.5 g/cm^3^ to avoid atomic overlaps. The COMPASS II force field was selected for all simulations [31]. This force field is a high-quality, Ab Initio-based parameter set specifically validated for condensed matter systems including polymers, organic molecules, and their composites, making it highly suitable for simulating SBSMA. The atom-based summation method with a cut-off distance of 15.5 Å was applied for van der Waals interactions, while the Ewald summation method was used for electrostatic interactions [31,43].
(3) Equilibrium Procedure (Annealing and NPT Ensemble): To obtain thermodynamically stable and representative models, a rigorous equilibration procedure was implemented.

Annealing: The initial models underwent an annealing process to sample a wider conformational space and escape deep local energy minima. This involved 10 cycles of molecular dynamics simulations between 298 K and 798 K (NVT ensemble, Nosé-Hoover thermostat), with each cycle lasting 100 ps. Between each high-temperature cycle, the system was quenched back to 298 K and subjected to further energy minimization (5000 iterations maximum) [44,45].

Equilibration: The annealed models were then subjected to a final equilibration run in the isothermal–isobaric (NPT) ensemble at 298 K and 1 atm for 500 ps. The Andersen thermostat and Berendsen barostat were used to maintain constant temperature and pressure, respectively. The timestep was set to 1 fs, and data was collected every 1000 steps. This step allows the system density to fluctuate and converge to its equilibrium value.

### 3.4. The Density SBSMA Molecular Models During the Kinetic Aging Process

**Figure 7:**
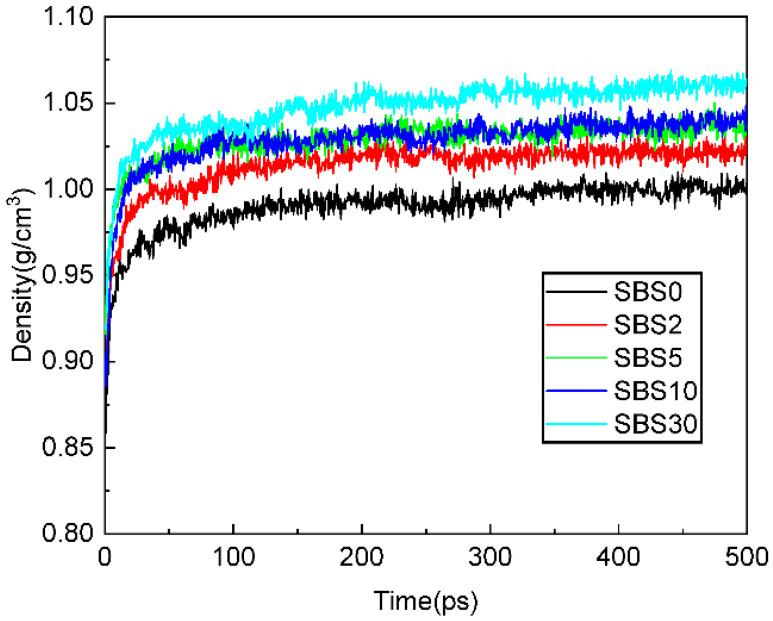
Densities of SBSMA molecular models’ different aging stages.

Figure 7 exhibits the simulated densities of various SBSMA molecular models, and these densities become stable with increasing simulation time after 100 ps. The densities of SBS0–SBS30 are obtained by calculating the average densities within 400–500 ps of each model, which are 1.003, 1.021, 1.038, 1.044 and 1.063 g/cm^3^, respectively. These densities are similar to those densities measured by previous studies [16,27], and are also in line with the density calculated through MD simulation in other studies [36,37,38]. Thus, the SBS0–SBS30 molecular models are reasonable. The average densities gradually increase over aging time. The first reason is that oxidative aging generates polar functional groups (carbonyl and sulfoxide) that enhance intermolecular interactions through stronger dipole–dipole forces and hydrogen bonding. These intensified interactions promote more compact molecular packing, directly contributing to density elevation. The second reason is that the compositional shift from lighter to heavier molecular components, as evidenced by SARA analysis, increases the overall mass concentration within the same volume. This transformation is particularly significant as aromatic fractions convert to resin and asphaltene components. The third reason is that the competitive absorption dynamics between asphaltene and SBS polymers play a crucial role. While both components initially absorb lightweight fractions—asphaltene for colloidal-structure stabilization and SBS for optimal swelling and performance transfer—the aging-induced breakdown of SBS’s three-dimensional network diminishes its absorption capacity. This shifts the balance toward greater asphaltene absorption, leading to tighter molecular packing and further density increase [8].

## 4. Simulation Results and Analysis

### 4.1. Cohesive Energy Density and Solubility Parameters During Aging

**Figure 8:**
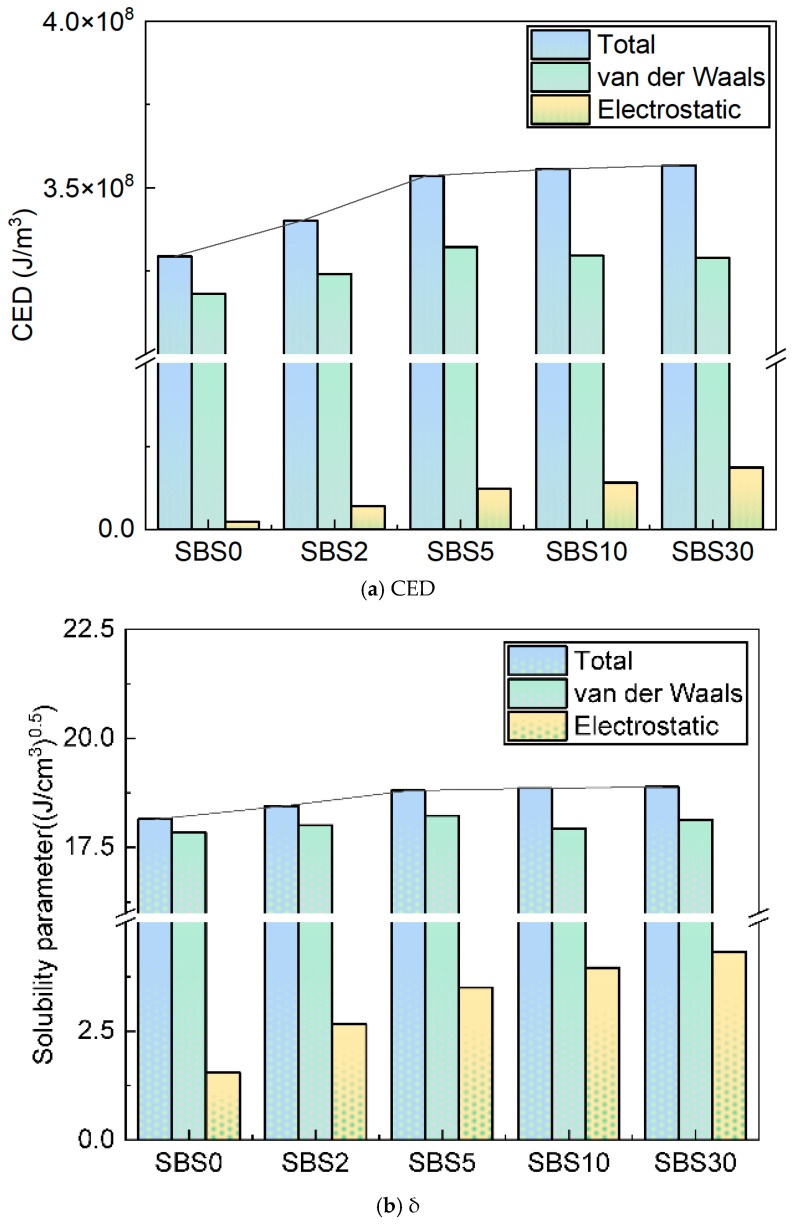
CED and δ of SBSMA molecular models during the kinetic aging process. (**a**) CED, (**b**) δ.

The aging-induced changes in cohesive energy density (CED) and solubility parameters (δ) of SBSMA reveal fundamental alterations in molecular interactions and system compatibility (Figure 8). The CED, representing the energy required for complete molecular separation [27], exhibits a characteristic biphasic growth pattern that aligns with the established aging kinetics. Throughout the 30-day aging period, the total CED increases, with van der Waals interactions accounting for a significant proportion increase, while electrostatic contributions, though initially negligible, become discernible in later stages.

The first aging stage (0–5 days) demonstrates a rapid CED increase of 18.4%, primarily driven by the formation of highly polar sulfoxide groups and the accelerated scission of SBS chains, which generates terminal polar groups (-OH, -COOH). These chemical transformations significantly enhance dipole–dipole interactions and hydrogen bonding within the system. In contrast, the subsequent aging stage (5–30 days) shows a more gradual CED increase, characterized by slower carbonyl-group formation and progressive molecular rearrangement as the broken polymer chains stabilize within the evolving SBSMA matrix.

Concurrently, the solubility parameter (δ) increases, reflecting a systematic deterioration in system compatibility [46]. This trend originates from three interrelated molecular-scale phenomena: the progressive polarization of the asphalt matrix through increased carbonyl and sulfoxide content coupled with asphaltene-fraction growth; the substantial degradation of SBS polymers evidenced by reductions in molecular weight and decreases in crosslink density; and the fundamental structural reorganization that weakens interfacial interactions between the polymer and asphalt matrix.

The CED and δ evolution suggest that compatibility reduction is principally governed by the intensification of intermolecular forces rather than simple compositional changes. These findings provide critical molecular-level insights into the aging mechanisms of SBSMA.

### 4.2. Surface Free Energy

The surface free energy (SFE) [47,48] of SBSMA at different aging stages were calculated based on their bulk model and confined model separately, according to Equation (5),

(5) $$\gamma_{a}=\left(E_{surface}-E_{bulk}\right)/2A$$

where $\gamma_{a}$ is the SFE, $E_{surface}$ is the potential energy of SBSMA molecular confined model, $E_{bulk}$ is the potential energy of SBSMA molecular bulk model, and A is the created new surface area.

**Figure 9:**
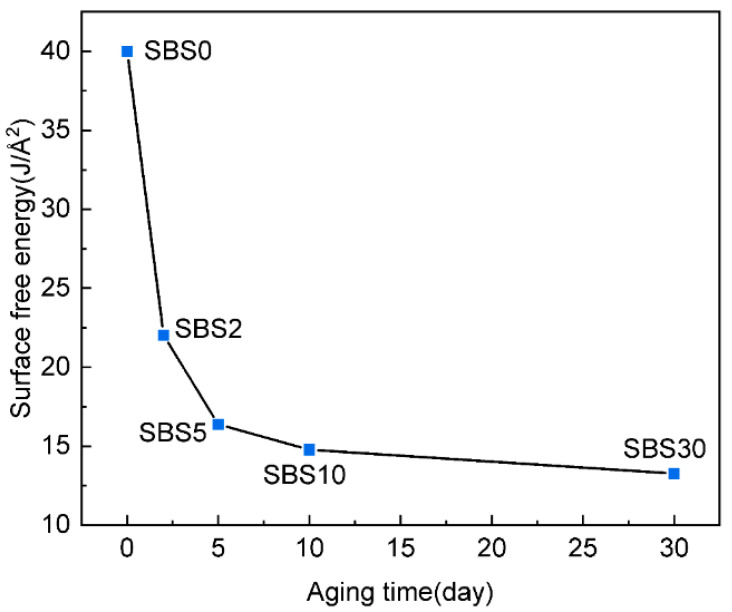
The surface free energy of SBSMA during the kinetic aging process.

Figure 9 illustrates the SFE of SBSMA during the kinetic aging process. The SFE exhibits a continuous decrease with aging time, indicating a reduction in the energy barrier for surface formation within SBSMA. This progressive decline in SFE over the 30-day aging period directly correlates with the SBSMA’s increasing susceptibility to cracking [28,46]. The reduced surface-energy barrier facilitates molecular separation, thereby weakening the internal adhesion of SBSMA and compromising its mechanical integrity. The aging process displays distinct kinetic phases in terms of SFE reduction: first aging stage (0–5 days), with rapid SFE decrease; and second aging stage (5–30 days), with gradual SFE decline.

### 4.3. Free Volume

The fractional free volume (FFV) was employed as a key metric to quantify free volume changes in SBSMA throughout the aging process, and was calculated according to Equation (6),

(6) $$FFV=\frac{V_{f}}{V_{0}+V_{f}}\times 100\%$$

where V_f_ is the free volume of SBSMA molecules and V_0_ is the occupied volume of SBSMA molecules.

**Figure 10:**
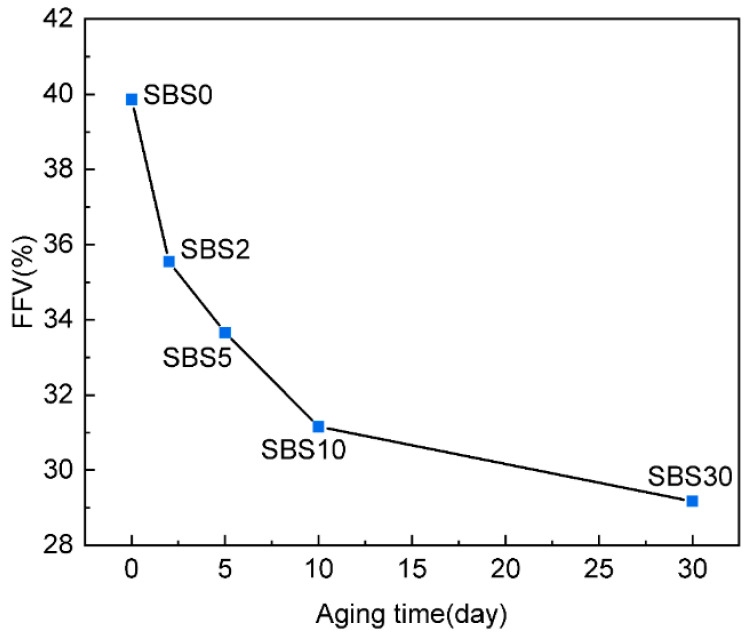
FFV of SBSMA during the kinetic aging process.

Figure 10 shows the changes in FFV of SBSMA during the aging kinetic process. It demonstrates a progressive reduction in FFV over the 30-day aging period, indicating substantial microstructural compaction that correlates with the observed hardening behavior. The first reason is that the enhanced intermolecular interactions from oxidation products drive molecular aggregation, reducing available void space. The formation of polar functional groups (sulfoxide, carbonyl) increases interaction energies, promoting tighter molecular packing. The second reason is that incorporated oxygen atoms and polar components physically occupy free volume, with oxygen content increasing during aging. These newly introduced atoms and functional groups reduce the available space for molecular motion. The third reason is that the breakdown of SBS polymer chains alters component distribution, shifting the adsorption equilibrium toward asphaltene-dominated absorption. This transition from polymer-swollen to asphaltene-dominated structures decreases average intermolecular distances. It is worthy to emphasize that the FFV of SBSMA decreases rapidly from SBS0 to SBS5, and slowly from SBS5 to SBS30, due to the predominant formation of sulfoxide groups in the first stage and extensive SBS-chain scission during initial aging.

### 4.4. Binding Energy Between SBS and Asphalt-Matrix Molecules

The binding energy resulting from the interaction between SBS and asphalt-matrix molecules enhances the deformation resistance and stability of SBSMA. To investigate this interaction during the aging process, the binding energy was calculated using Equation (7),

(7) $$E_{\mathrm{binding}(\mathrm{SBS}\text{-}\mathrm{Asphalt})}=-E_{\mathrm{inter}\left(\mathrm{SBS}\text{-}\mathrm{Asphalt}\right)}=-[E_{\mathrm{tol}(\mathrm{SBS}\text{-}\mathrm{Asphalt})}-E_{\mathrm{SBS}}-E_{\mathrm{Asphalt}}]$$

where E_binding(SBS-Asphalt)_ and E_inter(SBS-Asphalt)_ are the binding energy and interaction energy between SBS and asphalt-matrix molecules. E_tol(SBS-Asphalt)_ is the total energy of SBSMA molecules. E_SBS_ and E_Asphalt_ are the energy of SBS and asphalt molecules. The calculation of E_binding(SBS-Asphalt)_ process is as follows: (1) Computing E_tol(SBS-Asphalt)_ for the SBSMA model. (2) Removing SBS to determine E_Asphalt_. (3) Removing asphalt to calculate E_SBS_. (4) Deriving Ebinding_(SBS-Asphalt)_ via Equation (7).

**Figure 11:**
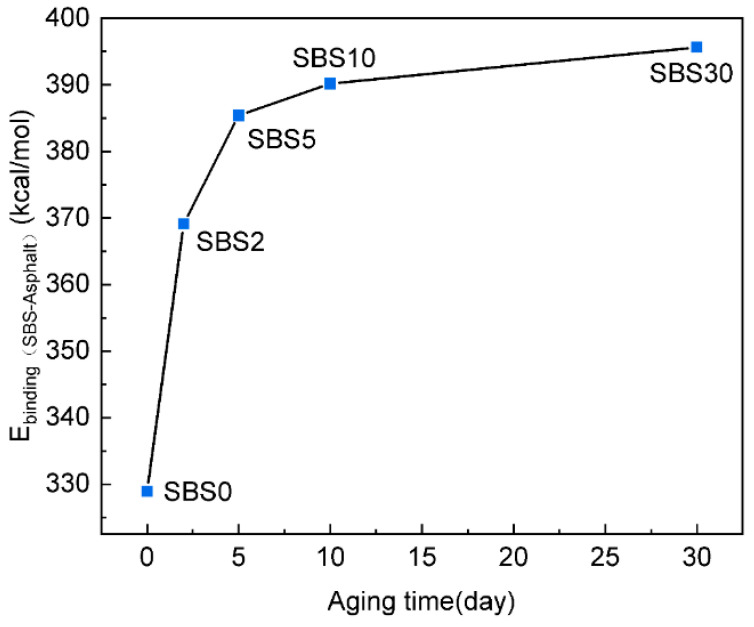
Binding energy of SBSMA during the kinetic aging process.

As shown in Figure 11, the binding energy increases with aging time, indicating stronger SBS and asphalt-matrix molecules interactions. Additionally, chain scission in SBS reduces its swelling capacity, limiting contact with the asphalt matrix. However, fragmented SBS molecules reorganize into tighter, more stable configurations, altering interaction between SBS and asphalt-matrix molecules.

It also appears that the binding energy increases rapidly from SBS0 to SBS5, and then slowly from SBS5 to SBS30. Additionally, the slower degradation of SBS chains during this stage delays the displacement of polymer fragments, leading to a more gradual increase in binding energy.

### 4.5. Radial Distribution Function

**Figure 12:**
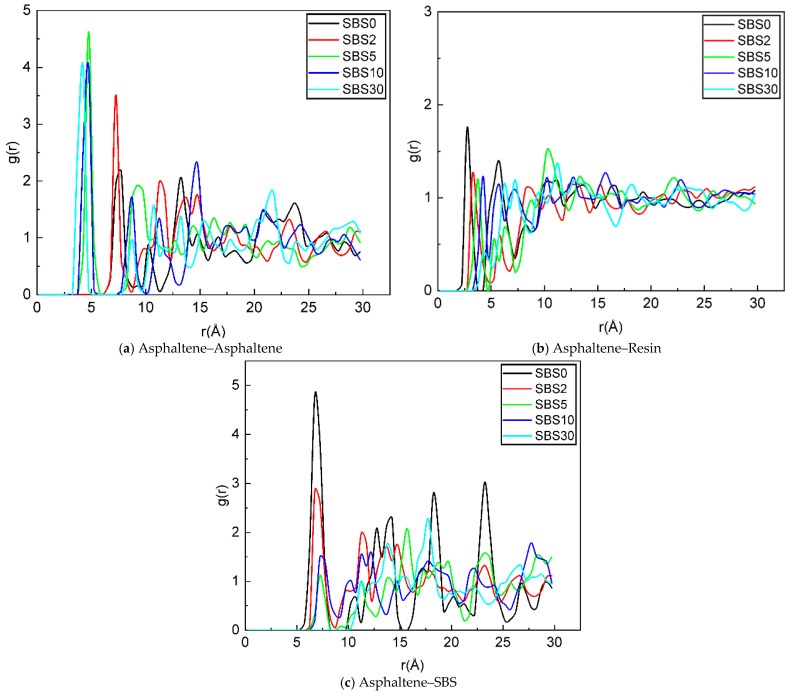
RDF of different components in SBSMA during the kinetic aging process.

To understand the colloidal-structure changes in SBSMA during the kinetic kinetic process, radial distribution function (RDF) was employed to characterize interactions among components, as depicted in Figure 12. The first peak distance for asphaltene–asphaltene pairs exhibits a gradual reduction with aging time. During the rapid aging stage (0–5 days), this distance sharply decreases from 7.75 Å to 4.75 Å, while in the slow aging stage (5–30 days), it shows a more gradual decline from 4.75 Å to 3.75 Å. This two-phase reduction pattern correlates strongly with the formation of different polar functional groups during aging. The initial rapid decrease is attributed to the generation of highly polar sulfoxide groups, which significantly enhances asphaltene aggregation. In contrast, the subsequent slower reduction phase corresponds to the formation of less-polar carbonyl groups, resulting in weaker intermolecular interactions.

Comparative analysis of Figure 12a,b reveals that asphaltene–asphaltene interactions consistently exhibit stronger self-aggregation tendencies than asphaltene–resin interactions across all aging stages. Notably, the asphaltene–resin distance increases from 2.75 Å to 6.25 Å during the entire aging period (SBS0-SBS30), indicating a progressive deterioration in their compatibility. This phenomenon suggests that enhanced asphaltene self-aggregation during aging disrupts the original colloidal structure, leading to the gradual detachment of resin molecules from asphaltene surfaces.

Furthermore, Figure 12c demonstrates a substantial increase in asphaltene–SBS distance from 6.25 Å to 11.25 Å throughout the aging process. Although SBS molecules initially maintain a stable distribution within the asphalt matrix due to their unique molecular structure, this stability is compromised by two concurrent aging effects: the fragmentation of SBS polymer chains and the intensified self-aggregation of asphaltene molecules. These combined processes ultimately lead to significant phase separation between the SBS and asphaltene components in the aged SBSMA system.

### 4.6. Relative Concentration

**Figure 13:**
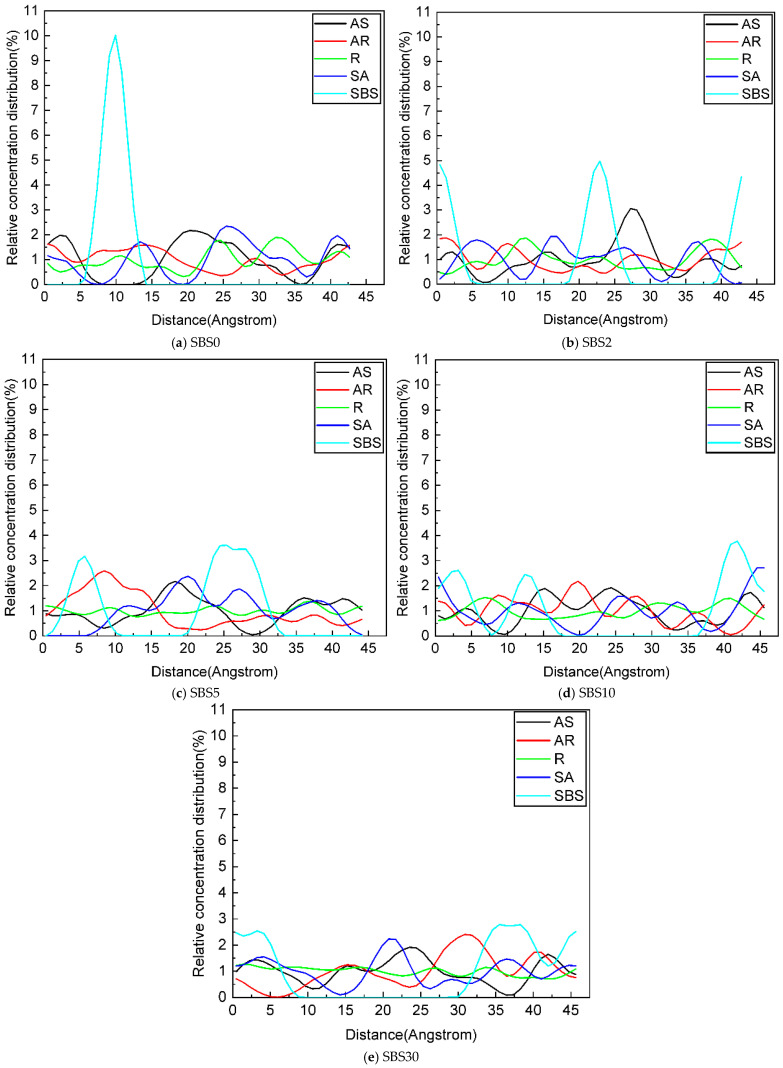
Relative concentrations of each component in SBSMA during the kinetic aging process.

The relative concentration distribution along the Z-axis (0,0,1) direction was analyzed to elucidate the dynamic redistribution of components during SBSMA aging, as presented in Figure 13. The unaged SBSMA (SBS0) exhibits distinct molecular organization, characterized by a prominent SBS peak at 10 Å, reflecting its well-defined aggregated state. Other components display different distribution patterns: asphaltene and resin show localized concentrations while aromatics maintain a homogeneous distribution, suggesting an initial equilibrium in the colloidal system.

The first aging stage (SBS2–SBS5) marks a transitional period where the molecular architecture begins to destabilize. The diminishing SBS peak intensity directly correlates with progressive polymer degradation, while the converging asphaltene and SBS peaks reveal emerging competitive interactions. This period is particularly significant as the rapidly evolving chemical environment, marked by increasing asphaltene polarity, reshapes the adsorption dynamics between components.

During the prolonged second-aging stage (SBS10–SBS30), the system undergoes more profound reorganization. The continuing decline in SBS peaks underscores the persistent breakdown of polymer integrity, while the pronounced accumulation of lighter fractions around asphaltene domains demonstrates a clear shift in adsorption preference. This transition to asphaltene-dominated adsorption arises from two synergistic factors: the cumulative effect of ongoing asphaltene and the corresponding loss of SBS functionality due to chain scission.

### 4.7. Diffusion Coefficient

**Figure 14:**
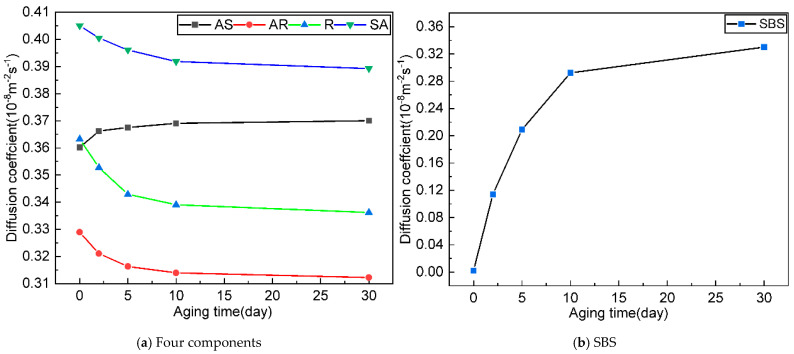
Diffusion coefficients each component in SBSMA during the kinetic aging process.

The dynamic changes in each component’s molecular mobility during SBSMA aging were quantitatively characterized through diffusion coefficient analysis derived from mean squared displacement (MSD) measurements, as presented in Figure 14. The results reveal a complex evolution of component mobility that directly reflects the microstructural transformations occurring during the aging process.

The diffusion behavior demonstrates a clear divergence among components. Asphaltene and SBS exhibit progressively increasing mobility with aging time, while resin and aromatic components show the opposite trend of decreasing diffusivity. This contrasting behavior stems from fundamental changes in molecular interactions and composition. The growing asphaltene content, resulting from aging-induced molecular conversions, leads to greater numbers of freely mobile asphaltene molecules, thereby increasing their overall diffusion coefficient. Simultaneously, the scission of SBS molecular chains reduces their capacity to adsorb lighter components, resulting in enhanced polymer-chain mobility as aging progresses.

Conversely, the decreasing mobility of resin and aromatic components reflects the strengthening of intermolecular interactions during aging. The increasing polarity and content of asphaltene enhances its adsorption capacity, causing more resin molecules to become tightly bound to asphaltene aggregates. Similarly, aromatic molecules experience restricted mobility due to both their conversion to heavier fractions and increased adsorption by the growing asphaltene phase. Even saturate components, despite maintaining relatively constant content, exhibit reduced mobility as they become increasingly incorporated into the developing asphaltene-dominated structure.

The aging process occurs through two distinct kinetic regimes characterized by different rates of mobility change. The initial rapid phase (0–5 days) features dramatic changes in diffusion coefficients, driven by the fast formation of highly polar sulfoxide groups and rapid SBS-chain scission. This is followed by a more gradual evolution phase (5–30 days) where mobility changes occur at reduced rates, corresponding to the slower generation of less-polar carbonyl groups and more gradual polymer degradation.

## 5. Conclusions

The comprehensive investigation of SBSMA aging reveals several key findings regarding its kinetic behavior and structural evolution:

(1) The aging process exhibits distinct two-stage kinetics. SI and CI demonstrate rapid increases during the initial 0–5 day period, followed by stabilization of SI and gradual CI growth from 5 to 30 days, indicating faster oxidation rates in the first aging stage. Similarly, the significant initial decrease in IS and IB within 0–5 days, followed by slower changes, confirms that SBS-molecular-chain scission predominantly occurs during the first aging phase.
(2) CED and δ analysis show that van der Waals interactions dominate the aging process. The faster CED increase during the first 0–5 days correlates with the formation of highly polar sulfoxide groups, compared to the less-polar ketones generated later. This initial period also features rapid SBS-chain scission, producing reactive hydroxyl and carboxyl groups that significantly enhance intermolecular interactions. Parallel trends in δ evolution and reductions in surface free energy, fractional free volume (FFV), and binding energies further confirm the first stage as the primary period of molecular reorganization.
(3) Radial distribution function analysis reveals important structural changes: asphaltene–asphaltene distances decrease more rapidly in the first aging stage due to stronger sulfoxide-induced aggregation. Conversely, increasing asphaltene–resin and asphaltene–SBS distances reflect declining compatibility from asphaltene self-aggregation and SBS-chain scission, ultimately destabilizing the colloidal structure.
(4) Component distribution analysis shows an evolution from initially dispersed SARA components and aggregated SBS to increasingly competitive adsorption behavior. The first aging stage establishes significant asphaltene–SBS competition, while the second stage demonstrates clear asphaltene dominance in component adsorption.
(5) Diffusion coefficient measurements reveal opposing mobility trends: asphaltene and SBS show accelerated mobility during rapid first-stage aging (0–5 days), while resin, aromatic, and saturate components exhibit decreased diffusivity. These trends moderate during the second stage (5–30 days).

## Figures and Tables

**Table 1:** The technical indices of asphalt matrix and SBSMA.

Properties	Test Results of Base Asphalt	Technical Standards	Test Results of SBSMA	Technical Standards	Test Method
Penetration (25 °C, 0.1 mm)	69.7	60–80	58.9	30–60	T0604-2011
Ductility (15 °C, cm)	>10	≥10	22.1	≥20	T0605-2011
Softening Point (°C)	>43	≥43	76.3	≥60	T0606-2011

**Table 2:** Assignment of major FTIR absorption bands in SBSMA spectra.

Wavenumber (cm^−1^)	Vibration Mode	Assignment
~2953	C-H asymmetric stretch	Aliphatic CH_2_ in asphalt and SBS
~2851	C-H symmetric stretch	Aliphatic CH_2_ in asphalt and SBS
~1700	C=O stretch	Carbonyl groups (products of oxidation)
~1600	C=C stretch	Aromatic rings in asphalt and polystyrene
~1542	N-O stretch/C=C stretch	Nitro compounds/Conjugated systems
~1460	C-H bending	Aliphatic CH_2_/CH_3_ in asphalt and SBS
~1376	C-H bending	Aliphatic CH_3_ in asphalt
~1199	C-O/S-O stretch	Esters, Sulfonic acids
~1086	S=O stretch	Sulfones
~1030	S=O stretch	Sulfoxide groups (products of oxidation)
~966	C=C-H bend	Trans-polybutadiene segment in SBS
~699	C-H bend	Monosubstituted benzene ring (polystyrene)

**Table 3:** The molecular numbers of SBS0–SBS30.

Model Classification	Molecular Name	Molecular Formula	Number of Molecules of SBS0	Number of Molecules of SBS2	Number of Molecules of SBS5	Number of Molecules of SBS10	Number of Molecules of SBS30
Aromatic	Dioctyl-cyclohexane-Naphthalene (DOCHN)	C_30_H_46_	16	12	10	8	8
Aromatic	Perhydrophenanthrene-naphthalene (PHPN)	C_35_H_44_	18	14	12	12	10
Asphaltene	Phenol	C_42_H_54_O	5	5	5	6	6
Asphaltene	Pyrrole	C_66_H_81_N	3	3	3	4	4
Asphaltene	Thiophene	C_51_H_62_S	5	6	6	6	6
Resin	Benzobisbenzothiophene	C_18_H_10_S_2_	5	6	8	10	10
Resin	Pyridinohopane	C_36_H_57_N	5	6	8	10	10
Resin	Quinolinohopane	C_40_H_59_N	20	21	22	23	24
Resin	Thio-isorenieratane	C_40_H_60_S	6	8	10	11	12
Resin	Trimethybenzeneoxane	C_29_H_50_O	7	9	12	13	14
Saturate	Hopane	C_35_H_62_	5	5	6	7	7
Saturate	Squalane	C_30_H_62_	5	5	6	7	7
	SBS	C_158_H_163_	1	-	-	-	-
	Broken SBS I	C_22_H_24_O	1	1	1	1	1
	Residual SBS I	C_136_H_141_O	1	1	-	-	-
	Broken SBS II	C_29_H_32_O	1	-	1	1	1
	Residual SBS II	C_107_H_112_O_2_	1	-	1	-	-
	Broken SBS III	C_53_H_58_O_3_	1	-	-	1	1
	Broken SBS IV	C_54_H_57_O	1	-	-	1	1

Note: Broken SBSI to SBSIV represent progressive fragmentation patterns of SBS.

## Data Availability

The original contributions presented in this study are included in the article. Further inquiries can be directed to the corresponding author.
